# Formulation approaches to pediatric oral drug delivery: benefits and limitations of current platforms

**DOI:** 10.1517/17425247.2015.1060218

**Published:** 2015-07-13

**Authors:** Felipe L Lopez, Terry B Ernest, Catherine Tuleu, Mine Orlu Gul

**Affiliations:** ^a^ University College London, School of Pharmacy, Department of Pharmaceutics, 29-39 Brunswick Square, London WC1N 1AX, UKfelipe.lopez.13@ucl.ac.uk; ^b^ GlaxoSmithKline, Product Development, New Frontiers Science Park, Third Avenue, Harlow, Essex CM19 5AW, UK

**Keywords:** acceptability, age-appropriate, formulation development, oral drug delivery system, pediatric drug delivery, technology platform

## Abstract

***Introduction*:** Most conventional drug delivery systems are not acceptable for pediatric patients as they differ in their developmental status and dosing requirements from other subsets of the population. Technology platforms are required to aid the development of age-appropriate medicines to maximize patient acceptability while maintaining safety, efficacy, accessibility and affordability.

***Areas covered:*** The current approaches and novel developments in the field of age-appropriate drug delivery for pediatric patients are critically discussed including patient-centric formulations, administration devices and packaging systems.

***Expert opinion:*** Despite the incentives provided by recent regulatory modifications and the efforts of formulation scientists, there is still a need for implementation of pharmaceutical technologies that enable the manufacture of licensed age-appropriate formulations. Harmonization of endeavors from regulators, industry and academia by sharing learning associated with data obtained from pediatric investigation plans, product development pathways and scientific projects would be the way forward to speed up bench-to-market age appropriate formulation development. A collaborative approach will benefit not only pediatrics, but other patient populations such as geriatrics would also benefit from an accelerated patient-centric approach to drug delivery.

## 1. Introduction

Pediatric patients require different oral drug delivery systems than other subsets of the population due to their continuing development hence dosing and administration requirements [1]. Conventional formulations are not designed for this patient group; thus, manipulation and compounding has become common practice [2]. Age-appropriate oral drug delivery systems specifically developed to meet the needs of the pediatric population are therefore desired. In terms of adherence and concordance geriatric patients would also benefit from patient-centric formulation design tailored to overcome the impaired physiological, visual, motoric functions and swallowing capabilities.

The development of an age-appropriate formulation is a challenging task due to the broad range of pharmaceutical and clinical aspects that must be considered in order to ensure the quality, safety and efficacy of the final product. In particular, the development of pediatric formulations is complex due to the additional needs and demands of this target population with respect to adults. The pharmacokinetic and pharmacodynamic profile of a drug varies broadly depending on the developmental stage of a child, necessitating dose flexibility to suit the dosing requirements across all age groups [3]. Excipients commonly regarded as safe may represent a safety risk for children adding other considerations into the formulation development [4]. Palatability and ease of swallowing are also considered as critical attribute for the acceptability of medicines intended for children, who possess distinct preferences and swallowing abilities than other subsets of the population. In many cases, the dependence on caregivers also influences the administration and acceptability of medicines [5].

In addition to all the factors mentioned above there are manufacturing, processing and packaging aspects to bring into the equation. The manufacturing process of pharmaceutical products must be robust and able to deliver medicines of adequate quality at an affordable price. Packaging and administration devices must be seen as an integral part of the product as these can improve the overall quality and acceptability of the medication [6,7], while minimizing its cost. The affordability of medicines is crucial for the development of pharmaceutical products for global market, including developing countries [8]. The utilization of cost-effective and readily-available technologies is often desired to maximize the affordability and accessibility of medicines, which ultimately benefits healthcare providers and patients. Therefore balance between innovative technologies and patient access to medicines must be sought.

An ideal formulation must gather a number of requirements to meet with the needs of patients, caregivers, manufacturers and healthcare providers. The numerous criteria that must be considered along the development of age-appropriate products has been classified into three main categories: i) factors related to efficacy and ease of use; ii) those related to patient safety; and iii) factors influencing the access of patients to medicines, as detailed in (Table 1) [9]. Considering the number of parameters that needs to be fulfilled, one single formulation development approach is less likely to be appropriate for all patients. Thus flexible technology platforms are desired enabling the preparation of formulations with different active pharmaceutical ingredients (APIs), dose strengths and/or release profiles [1,10].

**Table 1.** **List of requirements for age-appropriate oral drug delivery systems.**

**Benefit/risk**	**Criterion for drug product**	**Product requirements**
Efficacy/acceptability	Dosage	Dose flexibility Acceptability of size/volume
	Preparation/administration	Easy and convenient handling Easily administered (correct use)
	Compliance	Minimal impact on lifestyle Acceptable appearance and taste Minimal administration frequency
Patient safety	Bioavailability	Adequate bioavailability
	Excipients	Minimal number of excipients Tolerability
	Stability	Stable during shell life Stable in-use
	Medication error	Minimal risk of dosing error
Patient access	Manufacturability	Robust manufacturing process Commercial viability
	Affordability	Acceptable cost to patient and payers Easily transported and stored Low environmental impact

Data taken from [9].

**Figure 1.**
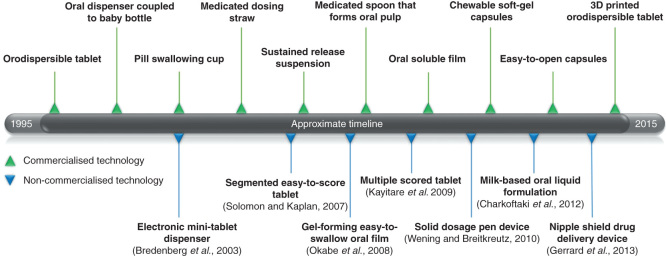
**A range of formulations and devices for age-appropriate oral drug delivery, which have emerged during the past two decades. **Green triangles above: commercialized dosage forms and devices; Blue triangles below: non-commercialized dosage forms and devices. This is not intended to be an exhaustive list but exemplify progress.

In recent years there has been an increased focus on the development of novel technologies for the preparation of age-appropriate formulations, supported by modifications in the regulatory framework [11]. This has resulted in a noticeable increase in the formulation design approaches (e.g., dispersible tablets, oral films and minitablets) and administration/dosing devices (e.g., medicated straw and minitablets dispensers) that has been investigated, patented and commercialized. Examples of technologies that have emerged during the past two decades are illustrated in (Figure 1). In this article, the current strategies for the development of oral drug delivery systems for pediatric patients are reviewed and their benefit and limitations critically discussed. The main focus of this work lay on marketed products and technologies as well as those close to market.

## 2. Recent advances in conventional oral drug delivery systems

Conventional solid (tablets and capsules) as well as liquid (solutions and suspensions) dosage forms exhibit limitations for the delivery of drugs to pediatric patients. In this section particular barriers for manufacturability and patient administration are discussed and recent developments to overcome existing limitations are reviewed.

### 2.1 Liquid dosage forms

Due to the inherent limitations of liquid dosage forms with respect to solid dosage forms (e.g., stability issues, challenging controlled release or higher transportation costs) the efforts of formulation scientists have been directed towards the development of solid formulations over liquids. However, liquid dosage forms may be favorable for certain patients (e.g., neonates and infants) due to the increased dose flexibility and ease of swallowing in comparison to solid products. Current developments have been focused on the design of dry solid formulations to be converted to liquid at the point of administration.

One of the major limitations of liquid products with regard to patient acceptability is the lack of controlled release formulations resulting in the need to administer multiple doses throughout the day. A number of approaches have been investigated for the development of sustained release liquids, such as ion exchange resins, coated microparticles in suspension or drug microemulsions, among others [12-14]. The relative success of each of these approaches is controversial. Nevertheless, few sustained release liquid formulations are available in the market such as azithromycin extended release (first extended release suspension) and methylphenidate hydrochloride extended release oral suspension [15-17].

**Figure 2.**
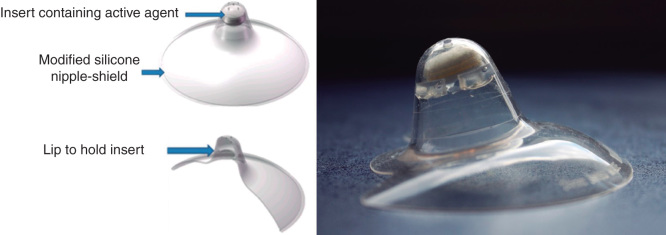
**Schematic illustration of the nipple shield device (left) and image of a prototype device including drug delivery insert (right).**

Reproduced from [20] with permission of Justmilk.org.

Recent work has been directed towards the investigation of appropriate vehicles for pediatric formulations with improved palatability. For example, milk has been explored as a vehicle in liquid formulations showing potential for solubilizing drugs while maintaining the stability of the emulsified vehicle [18,19]. The use of milk as a vehicle for the administration of drugs was also at the background of the development of a ‘nipple shield’ delivery system (Figure 2), which is designed to accommodate a drug-loaded insert delivering the API into milk while breastfeeding neonates [20,21]. Lipid-based vehicles are promising by providing solubilization of highly lipophilic drugs as well as masking the unpleasant taste [22]. Besides, self-emulsifying drug delivery systems can potentially be prepared as solid dosage forms for reconstitution [23].

An interesting growing field related to liquid dosage forms is the development of administration devices. Several dosing devices have been designed such as a baby bottle coupled to a syringe for aiding the administration of liquid formulations [24]. Others include modified pacifiers and the ‘dose sipping syringe,’ which can be used either as a conventional oral syringe or as a straw for the administration of liquid medicines [25,26]. The main potential limitation for wider applicability of these devices is the overall cost of the product.

### 2.2 Solid dosage forms

Solid drug delivery systems have been the formulation of choice for pharmaceutical industry due to the pros of well-established technology platforms enabling long-term stability, easing supply chain and maintaining low manufacturing cost. However, conventional solid forms may not be suitable for patients with swallowing difficulties, in particular for pediatric populations. Administration devices such as ‘pill swallowing cups’ have been used to increase the suitability of tablets and capsules of relatively large size to a broader population range [27]. However, acceptability studies are required to demonstrate the applicability of this type of devices in the most vulnerable populations with maximum need (e.g., infants). Additionally, education and training has proven to be a useful approach to facilitate swallowing of solid dosage forms [28].

Another limitation of conventional tablets is their poor flexibility of dose. Inevitably pill splitting has become usual daily practice to obtain various dose strengths. The use of ‘pill splitters’ is widespread despite the safety and efficacy risk of this practice [29,30]. In order to remove the risk, methodologies to improve the dose flexibility of single-unit dosage forms have been explored. Kayitare *et al*. developed a tablet that can be accurately scored into eight segments [31], whereas Solomon and Kaplan patented a novel technology for the preparation of tablets containing drug-free layers to aid accurate division without compromising the accuracy of the delivered dose [32]. An interesting development is the solid dosage pen, which consists of a cylindrical rod manufactured by mass-extrusion and incorporated into a pen-like device that allow dosing adjustments by cutting small tablet-like slices of the required length [33,34].

Smaller tablets and capsules emerge as an alternative to conventional solid dosage forms with improved dose flexibility hence ease of swallowing. Several studies have shown that young children from the age of 6 months are able to swallow single minitablets [35,36]. Moreover, 2 mm minitablets proved to be more acceptable than syrups even for the very young subgroups (6 – 12 months old) [36]. Nevertheless, the maximum dose that can be delivered by single-unit minitablets will always be limited by their small size. In consequence, several of these small-sized tablets are typically required in order to achieve the targeted dose. The administration of multiple minitablets is further discussed in the following section dedicated to multiparticulate drug delivery systems.

New packaging systems of solid dosage forms are also evolving with the aim of improving both the safety and the acceptability of medicines. Compliance-prompting packaging include printed blisters to facilitate self-monitoring of the treatment (calendar packaging) plus guidelines for correct administration which, in combination with education and other reminder strategies when needed, may improve medication adherence [37].

## 3. Novel approaches to age-appropriate oral drug delivery

In this section new formulation design approaches are reviewed, including multiparticulate drug delivery systems, orodispersible tablets (ODTs), orodispersible films (ODFs), and chewable formulations. The parameters listed in Table 1 are used as a guidance to critically discuss the advantages and limitations of each technology platform.

### 3.1 Multiparticulate drug delivery systems

Multiparticulate drug delivery systems are composed of a number of discrete units such as granules, pellets or minitablets. Multiparticulate products are expected to provide improved patient acceptability over single-unit solid dosage forms (i.e., tablets and capsules) by dint of their reduced size and thus improved ease of swallowing plus the increased dose flexibility provided by their multi-unit composition. Moreover, multiparticulate products are usually suitable for controlled release and taste masking by means of film-coating technologies, which can also benefit patient’s compliance. Characteristic advantages and limitations of multiparticulate drug delivery systems are summarized in Table 2.

**Table 2.** **Advantages and disadvantages of multiparticulates for the preparation of age-appropriate products.**

**Product characteristic**	**Advantages**	**Disadvantages**
*Efficacy/acceptability*		
Dosage	Excellent flexibility of dose Small size/swallowing is aided	Grittiness/mouthfeel may be an issue
Preparation	Flexibility of administration	Need for preparation/reconstitution
Compliance	Ease of functionalization Suitable for taste masking	
*Safety profile*		
Bioavailability	Highly reproducible due to uniform GI transit Targeted release profiles can be achieved	Co-administration with food/drinks may alter bioavailability
Excipients	Use of Generally Regarded As Safe (GRAS) excipients	
Stability		Food-drug compatibility needs to be studied
Medication error		Limited control over dose intake when mixed with food
*Patient access*		
Manufacturability		May need specialize equipment or accessories
Affordability	Manufacturing technology readily available	Need to develop packaging/dosing technology platform

Small particulates may be easier to swallow and thus more acceptable than single-unit formulations for certain populations. However, the acceptability of multiparticulates in terms of grittiness or mouthfeel is not fully understood [38,39], possibly limiting the development of these products. There is also a lack of evidence on the size and amount of multiparticulates that is acceptable to patients, although recent FDA guidance suggests a maximum targeted size of 2.5 mm [40]. Research is required in this area, where the utilization of robust predictive models to assess palatability is desired as it could avoid the hurdles of conducting clinical trials [41]. Meanwhile, oral gels and *in situ* gelling vehicles are being studied as media to aid the administration of multiparticulate formulations [42]. Multiparticulates can be directly administered into the patients’ mouth or dispersed in a vehicle prior to administration as preferred. Water, milk, juice or apple sauce are potential vehicles commonly proposed [43]. The administration of multiparticulates in admixture with food (‘sprinkling’) is often indicated to improve the organoleptic properties and thus the acceptability of these formulations. However, despite of the potential to improve palatability, the need for product preparation may actually have a negative impact on the overall acceptability of the product as shown in recent studies [44,45]. In addition, the co-administration of drug products with food or drinks causes safety concerns, such as poor control over dose intake and impact on drug’s bioavailability [46]. Therefore, the influence of this practice on the product safety and efficacy should be considered beforehand. In this respect Albertini *et al*. investigated the compatibility of solid lipid microparticles in milk and yogurt as suitable vehicles for pediatric administration [47]. In any case, the need for product manipulation by the patient or caregiver should always be kept to a minimum.

The multi-unit composition of multiparticulate drug delivery systems offers attractive opportunities for the preparation of fixed-dose combinations and products with targeted release profiles, which can reduce the burden of repeated administration [48]. This can be achieved by simply combining multiparticulates with different APIs and/or different release characteristics into the same dosage form, respectively. An advantage of multiparticulates over single-unit formulations is that controlled release and thus improved bioavailability can be provided while avoiding the risk of dose-dumping [49]. In addition, multiparticulate products have been reported to provide a more reproducible distribution in the gastro-intestinal tract with lower risk of local irritation, although knowledge in this field is still limited and subjected to a high degree of inter- and intra-individual variability [50].

There is a broad range of manufacturing techniques that can be used to prepare multiparticulate products, with extrusion-spheronization and active layering the most commonly reported. Other manufacturing methods for the preparation of multiparticulates include fluid bed granulation [51], spray-drying [52], and microencapsulation techniques [53,54]. As for production of adult medicines, single-step manufacturing (direct pelletization) is preferred over multi-step processes in order to reduce cost and variability [55]. All these technologies render spherical particulates of small diameter (typically < 1.5 mm). In addition, minitablets of 1 – 3 mm can be prepared by conventional tableting equipment, using either small conventional tooling or specialized accessories [56]. The production of mini-tablets is often more demanding than larger tablets and thus an excellent understanding and control of processing variables is needed and specialized excipients are often required in order to obtain the targeted flow and compression properties [57]. The manufacturing process of multiparticulate products usually include a polymeric coating step as downstream processing for improved aesthetical properties, taste masking and/or controlled release functionalization.

**Figure 3.**
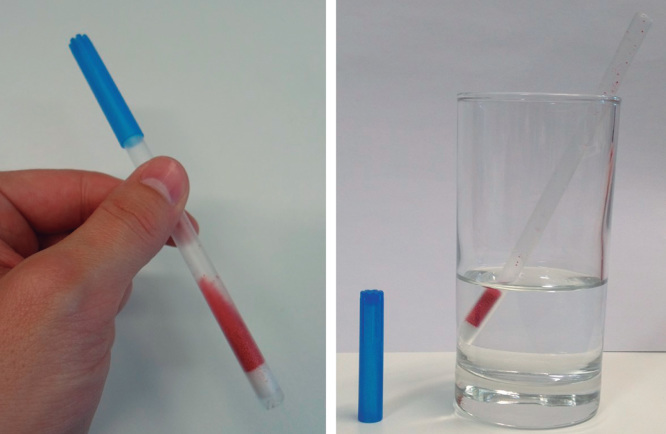
**Dose sipping technology: prototype straw containing granulated product with removable cap (left) and without cap, ready-to-use in a glass of water (right).**

Multiparticulates also offer a great degree of flexibility in terms of presentation and packaging. First, these formulations can be filled into capsules, although this could limit their swallowing advantage unless presented as an easy-to-open capsule to the patient [58]. In addition, multiparticulate products can be prepared as single-dose sachets, which allow for higher doses than tablets or capsules. Moreover, granules or pellets can be incorporated into medical devices to aid administration. This is the case of medicated spoons which contain a single-dose granulated formulation that can be designed to be either dispersed in a beverage prior to administration [59] or submerged in water to form an easy-to-administer pulp [25]. Another example of administration devices is the dose sipping technology in which a pre-dosed granulated medicine is filled into a ready-to-use straw (Figure 3) [26]. This technology reached the market for the oral delivery of the antibiotic clarithromycin but due to commercial pressures the availability of the product was limited after few years of commercialization.

The aforementioned approaches are intended as single-dose presentations, besides multi-dose presentations may also be considered providing further advantages in terms of dose flexibility. This would require the utilization of dosing devices to allow adjustment of the dose by measuring different amounts/volumes of multiparticulates from a pre-filled multi-dose pack. Research has been conducted in this direction and several patents have been filled with devices ranging from dosing spoons to electronic dispensers [7]. In general, volumetric spoons are the most cost-effective approach, although their success to achieve accurate dosing is limited (which is particularly important for drugs with a narrow therapeutic index). More sophisticated devices can lead to highly accurate dosing by counting, although these technologies may be more costly to develop and produce. The applicability of these devices to accommodate different formulations (potentially with a different size and/or shape) is desirable in order to reduce costs. More detailed information about devices for oral administration can be found in a recent review by Wening and Breitkreutz [7].

### 3.2 Orodispersible tablets

ODTs are designed to disintegrate in the oral cavity within a matter of seconds, avoiding the need for swallowing the tablet as a whole [60]. In some cases, when the disintegration/dissolution is sufficiently fast, the use of water can also be avoided. Moreover, ODTs offer great flexibility in terms of administration, as the tablet may be pre-dispersed in a suitable vehicle, dispersed directly in the mouth or even swallowed as a whole as preferred. Owing to these benefits, patients’ acceptability and compliance can be improved with respect to conventional formulations. The main characteristics of ODT formulations are summarized in Table 3 and are further discussed below.

**Table 3.** **Advantages and disadvantages of orodispersible tablets for the preparation of age-appropriate products.**

**Product characteristic**	**Advantages**	**Disadvantages**
*Efficacy/acceptability*		
Dosage		Various dosage strengths required
Preparation	Water is not required Swallowing is avoided Flexibility of administration	Lack of mechanical strength
Compliance	Preferred over conventional formulations	Controlled-release is challenging Taste masking is challenging
*Safety profile*		
Bioavailability	May be improved by buccal absorption	
Excipients		Excipients of unknown safety profile may be required
Stability		Packaging and storage conditions can be critical
Medication error		Retention time in mouth may alter bioavailability
*Patient access*		
Manufacturability		High doses may not be incorporated
Affordability		Technologies subjected to intellectual property rights

Although ODTs facilitate administration and swallowing, this formulation design do not bring an advantage in terms of dose flexibility with respect to conventional tablets, meaning that various dosing strengths would be required to fulfill the needs of all populations. In addition, owing to the fragility of ODT formulations, tablet splitting is usually contraindicated [15], which may further reduce dose flexibility. These limitations could potentially be overcome via preparation of ‘orally disintegrating minitablets’, an interesting opportunity to combine the benefits of ODTs and multiparticulates [61].

ODTs can be swallowed once disintegrate in the mouth to provide drug absorption mostly along the gastrointestinal tract or, alternatively, retained in the mouth for sublingual or buccal absorption, which may offer advantages in terms of onset of action and bioavailability for those drugs that can be absorbed through the oral mucosa. Formulations designed for buccal absorption may incorporate a bio-adhesive layer to facilitate retention of the formulation in the oral cavity and/or to target a particular absorption site inside the mouth [62]. The intended use of ODTs must be clearly stated to avoid medication errors as the formulations’ retention time in the mouth could potentially alter the bioavailability of the drug.

As the drug is subject to the patients’ taste buds in the mouth, taste masking is a requirement of orally disintegrating formulations with unpleasant tasting APIs. Improved palatability is traditionally achieved by addition of sweeteners and flavors to the formulation. However, the efficacy of this approach is often limited and, in addition, the use of these excipients poses safety concern (especially for pediatric patients) [63]. Coating of the drug particles represents an effective way of taste masking, however technologically more challenging [63,64]. Nevertheless, patented ODT technologies have been able to overcome this challenge through the preparation and subsequent compression of microencapsulated drugs for improved organoleptic properties and/or polymer-coated particles for customized release [65].

There are various approaches for the development of ODTs including lyophilization, direct compression, tablet molding, flash heat processing and lately 3D printing technology. Lyophilization and direct compression are by far the most commonly used manufacturing methods. In general terms, lyophilized tablets are mechanically more fragile than compressed ODTs and often require specialized packaging to ensure stability. Lyophilized ODTs are also limited by the maximum dose that can be delivered, usually < 400 mg for poorly water-soluble drugs and down to ∼ 60 mg for water-soluble drugs [66]. In return, lyophilized ODTs offer quicker disintegration (often <10 s) than tablets prepared by compression. Moreover, the formulation development of compressed ODTs is usually tedious, as it is challenging to get the right balance between quick disintegration and appropriate mechanical strength [66]. The relative benefits and disadvantages of the different manufacturing approaches for the development of ODTs have been widely discussed in the past; the interested reader is thus referred to previous reviews of this topic [66-69].

The production of ODTs is highly controlled by patented technologies. Fast dissolving technology based on a continuous ‘form-fill-freeze’ process in which doses deposited in blisters are lyophilized has been the leading technology in ODTs [70]. Other ODT technologies have been built on lyophilization or compression proprietary manufacturing processes and branded under different trade names [71]. A very recent ODT platform is based on 3D printing, which enables the preparation of ‘sponge-like tablets’ with high drug loading (up to 1000 mg) and very rapid disintegration (< 10 s), overcoming some of the limitations of both compressed and lyophilized ODTs [72].

Despite of the costs derived from the development and production of ODTs, often subjected to manufacturing and/or packaging processes that are costly and controlled by intellectual property rights, the number of ODT products in the market is rising considerably. Although most of these products are recommended for adolescents and adults, an increasing amount of pediatric ODT formulations are also available for younger children. For example, a recently marketed ODT is recommended for children as young as 1 year old; the formulation can be directly administered into the patient’s mouth or, alternatively, dissolved in water for administration via either an oral syringe or a nasogastric tube [73].

### 3.3 Orodispersible films

Drug-loaded ODFs based on polymeric matrices can be designed to disintegrate quickly in the mouth releasing the active ingredient. Swallowing is aided by the quick disintegration/dissolution of ODFs in the oral cavity in a similar fashion to their predecessor ODTs, eliminating the need of water for their administration. Moreover, ODFs possess an elegant appearance and may be preferred by some patients. An added benefit of films in comparison to tablets is their increased flexibility of dose, as different strengths can be achieved by simply cutting films of the required size [74]. A comprehensive list of advantages and disadvantages of ODFs is provided in Table 4.

**Table 4.** **Advantages and disadvantages of orodispersible films for the preparation of age-appropriate products.**

**Product characteristic**	**Advantages**	**Disadvantages**
*Efficacy/acceptability*		
Dosage	Excellent dose flexibility	
Preparation	Water is not required Swallowing is avoided	
Compliance	May be preferred over conventional formulations	Controlled-release is challenging Taste masking is challenging
*Safety profile*		
Bioavailability	May be improved by buccal absorption	
Excipients		Excipients of unknown safety profile may be required
Stability		Specialized packaging often required
Medication error		Retention time in mouth may alter bioavailability
*Patient access*		
Manufacturability	Continuous manufacturing can be achieved	Uniformity of dose may be challenging Only low doses can be incorporated
Affordability		Technologies subjected to intellectual property rights Solvent-based manufacturing process

An important limitation of ODFs is that taste masking and controlled release is technologically challenging. The utilization of coating techniques for these purposes is limited by the own nature of the manufacturing process, which usually involves solubilization of the API [74]. In some cases, sustained release has been achieved through the preparation of multi-layered films by combining layers with different release-controlling polymers. However, the fast-disintegrating advantage is not purposeful anymore as they are often designed to adhere onto the buccal mucosa and release the active ingredient in a timely manner. In addition, the absorption of drugs through the oral mucosa is limited and thus controlled release ODFs are often intended for topical delivery rather than systemic delivery of drugs [75].

ODFs are composed of a polymeric matrix with a drug embedded, typically manufactured by means of solvent casting method. By this method, a solution containing the active ingredient along with the film-forming polymer, plasticizer(s) and other required excipients is allowed to evaporate leaving a solid film behind. In some cases metering rollers can be used to determine the thickness of a wet mass which is subsequently dried and cut into pieces of appropriate size to achieve the desired dose [74]. Alternatively, ODFs can be prepared by hot-melt-extrusion where the use of solvents is avoided, offering potential benefits for controlled release and taste masking [76]. In addition, novel technologies for the preparation of ODFs are arising, such as electrospinning or ink-jet printing [77,78]. Regardless of the manufacturing method, the amount of drug that can be loaded in ODFs is very limited (typically < 60 – 70 mg [79]) owing the ODFs reduced size (2 – 9 cm^2^) and thickness (25 µm to 2 mm). Although novel technologies can incorporate higher drug doses of > 100 mg [80], this amount is still limited and thus only potent drugs with specific physicochemical properties can be successfully delivered [72].

**Figure 4.**
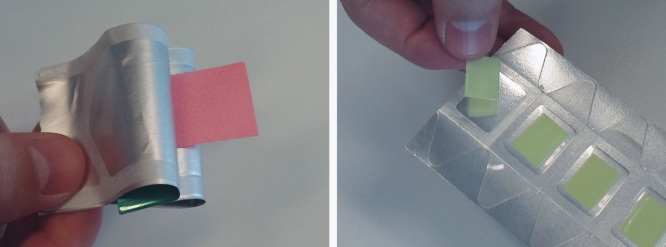
**Examples of orodispersible films in single-dose (left) and multiple-dose (right) packaging alternatives.**

ODFs are normally presented to the patient as stamp-like strips, either in single-dose sachets or contained in multi-dose packs (Figure 4). Preferably, ODFs should be sealed individually in order to improve stability and reduce the risk of overdosing due to films sticking together [74]. Potentially, more sophisticated multi-dose dispensers could be used where the desired dose is achieved by the patient or caregiver by cutting strips of appropriate length from a tape-like supply [81]. However, this approach incurs in higher development and production costs and may also increase the risk of dosing errors.

The need for specialize manufacturing and packaging equipment may reduce the viability of the ODF technologies. In fact, several commercially available ODF products have been discontinued in the past, manufacturing issues and poor revenue being potential factors behind the market discontinuation of these products [15]. Over-the-counter medicines lead the market of ODFs, including vitamins and food supplements, breath fresheners, antihistaminics and cough suppressants [79]. The first prescription-only ODF to reach the market was ondansetron oral-soluble film, indicated for adults and children from 4 years of age in USA [82].

### 3.4 Chewable formulations

Chewable formulations (i.e., chewable tablets, soft-chews and chewing gum) are designed to be mechanically processed in the mouth to aid disintegration and/or dissolution of the API. These products offer advantages for their administration in the sense that swallowing is aided (or avoided in the case of chewing gum) and water is not required. In addition, chewable dosage forms may be preferred by patients over other formulations due to their aesthetic properties. However, as in the case of ODTs, chewable products do not offer an advantage in terms of dose flexibility with respect to conventional tablets. The main advantages and limitations of chewable formulations for the administration of medicines to pediatric patients are summarized in Table 5.

**Table 5.** **Advantages and disadvantages of chewable tablets for the preparation of age-appropriate products.**

**Product characteristic**	**Advantages**	**Disadvantages**
*Efficacy/acceptability*		
Dosage		Various dosage strengths required
Preparation	Water is not required Swallowing is avoided	
Compliance	May be preferred over conventional formulations	Controlled-release is challenging Taste masking is challenging
*Safety profile*		
Bioavailability	May be improved by quick disintegration and dissolution May be improved by buccal absorption	Bioavailability may be altered depending on chewing ability
Excipients		Excipients of unknown safety profile may be required
Stability		Soft-chews may be problematic due to water content
Medication error		Retention time in mouth may alter bioavailability Possible overdose if misused as confectionary
*Patient access*		
Manufacturability		May need specialize equipment or accessories
Affordability	Manufacturing and packaging technology readily available	

Disintegration and swallowing of chewable dosage forms is aided by the patient by means of chewing and/or sucking. Therefore, taste and mouthfeel become critical attributes and thus a considerate decision should be made on the selection of excipients [83]. Sugar-based fillers and sweeteners such as mannitol, sucrose and sorbitol are often used to improve palatability. A particular disadvantage of chewable products is their poor suitability for taste masking and controlled release by coating techniques, as the formulation is subjected to a great mechanical stress upon administration. In addition, the drug release process and thus the therapeutic effect are dependent on the patient’s chewing ability, which may result in intra- and inter-individual variability.

The need for chewing of the dosage form may represent a limitation for the applicability of chewable dosage forms in the pediatric population. However, available data suggest that chewable tablets are safe and well-tolerated in children from 2 years of age [84]. As opposed to chewable tablets the gum-based core of chewing gums is not meant to be swallowed. For this reason, the time required to achieve complete dissolution of the API should be determined and stated in the product label. There is a lack of evidence about the safety of chewing gum in young children and current guidelines only recommend its use for children of 6 years or older [85]. Besides, concerns have been raised about the possible misused of these products which may be appreciated by children as confectionery [85].

Chewable tablets are typically prepared by compression in a similar fashion to compressed ODTs, but disintegrating agents are not included in the formulation. There are also patented technologies for the preparation of chewable formulations. For example, Paulsen *et al*. described a manufacturing method based on tablet molding where the use of water and elevated temperatures is avoided [86]. Other approaches are based on soft gelatine capsule technology modified by the addition of chewable filler, providing the benefits of soft-gels while avoiding the need for swallowing the capsule as a whole [87,88]. Pharmaceutical chewing gum is prepared by addition of artificial resins, waxes and elastomers to the formulation prior to compression or extrusion [89]. Gum-based tableting technology has been successfully applied for the local delivery of drugs such as fluoride and chlorhexidine [90].

## 4. Conclusion

The development of age-appropriate pharmaceutical products is challenging due to the combined demands of industry, healthcare providers, caregivers and patients. During the past two decades an important number of age-appropriate products have been investigated, developed and patented, and some have gained marketing authorization. The current strategies for the preparation of age-appropriate oral drug delivery systems have been reviewed throughout this manuscript.

Unfortunately, the limited information available regarding acceptability and patient preference of emerging dosage forms (i.e., ODTs, ODFs, chewable formulations, multiparticulates and minitablets) for the different age subgroups hinder the rational selection of one formulation approach over another. Owing the diversity of the pediatric population and the discussed limitations of the current technologies it seems unlikely that a single formulation approach will be acceptable for all pediatric patients. The selection of a suitable formulation approach for a targeted population group needs to be carefully considered for each individual product. Further investigation in this field is desired to allow correlation between formulation technological aspects and patient acceptability that guides such a selection process.

## 5. Expert opinion

In recent years there has been an important sum of efforts from regulators, industry and academia towards the development of patient-centric pharmaceutical products. This has resulted in a noticeable increase in the number of age-appropriate formulations available for some pediatric indications.

Some of the reviewed formulation approaches for the preparation of age-appropriate drug delivery systems are proving relative success. In particular, the ODT technology platform has been commonly visited by industry enable product line extension as well as addressing pediatric patient needs. ODFs are also becoming increasingly popular, although there are technical barriers that need to be overcome to broaden the spectrum of APIs and doses that can be delivered by ODFs. Meanwhile, pellets and minitablets offer potential alternatives for pediatric patients although there is still, even if encouraging, limited evidence to support their suitability for young children. Paradoxically, most of the multiparticulate products available in the market are filled/compressed into capsules/tablets restricting the benefits of multiparticulates for children such as ease of swallowing and dose flexibility. Besides, the investigation into devices for individualized dosing of multiparticulates has not reached out patients yet.

The regulatory incentives for the development of age-appropriate medicines have been a step forward in terms of increasing the number of authorized pediatric formulations [91]. Despite this promising increase, manipulation and compounding still continue to be common practice among caregivers. Therefore further strategies need to be developed to guide the research in the field prioritizing not only the design but also the feasibility and scalability of the manufacturing process to enable rapid translation of discoveries and patented technologies into marketed products in a cost-effective way. Collaboration between regulators, industry and academia should continue to evolve to facilitate the process ‘from bench to market’.

The selection of the most appropriate formulation design and excipients needs to be guided by a compendium of patient safety, manufacturability and end-user requirements (e.g., palatability and ease of use). Attempts have been made to define the most appropriate formulation for each particular patient subgroup [9,85]. However, there is still limited evidence-based data and thus lack of understanding of the effect of pharmaceutical technologic aspect on patient-related outcomes [92]. Patient acceptability should be considered at an early stage in the product development pathway rather than as a consequence of the formulation development process. The development of robust *in vitro* analytical tools to predict patient-related outcomes would be highly desirable to achieve this goal.

Flexible technology platforms are attractive for industry by enabling the delivery of multiple drugs, dose strengths and release profiles as well as being acceptable for broader patient populations. There are cases where age-appropriate formulations are not only favorable for children but also for other special patient groups including elderly and adults with reduced capability to swallow conventional solid formulations [93]. For example, multiparticulate and orodispersible formulations initially designed for pediatrics may be appropriate for others. Targeting a larger patient population may improve the commercial viability of pediatric products but caution must be taken to ensure that this practice does not undermine the requirements of each patient group. Further research is required to generate evidence-based data that support the utilization of a particular formulation in different age groups with/without an additional administration device.

Along with the implementation of technology platforms that enable the preparation of age-appropriate oral dosage forms, there are extemporaneous dispensing activities to achieve dose flexibility for the individual patient. For example, in Japan it is already common practice among pharmacies to prepare personalized medicines at the point of administration using small-scale packaging equipment to fill in sachets with the required dose of a granulated drug product [94]. Similar interim practices might be considered globally as long as the quality, efficacy and safety of the formulation are maintained. A balanced approach between innovation and cost-effectiveness must be sought to provide patient acceptability without impairing the access to patients of new medicinal products.

**Article highlights.**

- Age-appropriate oral formulations are expected to meet all the quality attributes of conventional pharmaceutical products as well as specific patient requirements (e.g., higher degree of dose flexibility and ease of swallowing).
- The most popular technologies to date have been for the manufacture of small-sized solid oral drug delivery systems (e.g., minitablets and multiparticulates) and orally dispersible products (e.g., orodispersible tablet, orodispersible films and chewable formulations).
- Age-appropriate administration devices (e.g., the solid dosage pen, multiparticulate counters and medicated straws) may improve the acceptability of pharmaceutical products.
- Not only pediatric patients but also geriatric patients and adults with reduced capability to take conventional solid formulations may benefit from patient-centric approaches to drug delivery.
- A balanced approach between innovation and cost-effectiveness is required to enable high-quality products while not-impairing patient access to better medicines.

This box summarizes key points contained in the article.
